# The Potential of MicroRNAs in the Context of Sarcopenic Obesity

**DOI:** 10.33549/physiolres.935755

**Published:** 2025-12-01

**Authors:** Nela CHOBOLOVÁ, Zdeněk ŠVAGERA, David STEJSKAL, Marek BUŽGA

**Affiliations:** 1 Institute of Laboratory Medicine, Faculty of Medicine, University of Ostrava, Ostrava, Czech Republic; 2 Institute of Laboratory Medicine, Department of Clinical Biochemistry, University Hospital Ostrava, Ostrava, Czech Republic; 3 Institute of Physiology and Pathophysiology, Faculty of Medicine, University of Ostrava, Ostrava, Czech Republic

**Keywords:** microRNA, Sarcopenic obesity, miRNA, Biomarkers, Muscle atrophy, Inflammation

## Abstract

Sarcopenic obesity (SO) is a complex pathological condition characterized by the simultaneous presence of excessive adipose tissue and the loss of muscle mass and strength. This combination leads to an increased risk of metabolic, cardiovascular, and functional complications. In recent years, there has been growing interest in the use of microRNAs (miRNA) as biomarkers capable of detecting early changes in body composition and predicting the progression of SO. MiRNAs are small noncoding RNA molecules that play a key role in regulating gene expression and cellular pathways related to inflammation, metabolism, and muscle trophism. This article summarizes current knowledge about miRNAs expression in patients with sarcopenic obesity, their regulatory functions, and their potential use in diagnostics and therapy.

## Introduction

During the past two decades, there has been a dramatic increase in the prevalence of obesity, which has become a serious global health issue with significant socioeconomic consequences. In countries in Central and Eastern Europe, including the Czech Republic, obesity is among the leading epidemiological threats [1]. According to the results of a large epidemiological study conducted in the Czech Republic, 30 % of the population is overweight and an additional 25 % is obese [2], significantly increasing the incidence of cardiometabolic diseases, functional impairments, and mortality.

Current therapeutic approaches to obesity involve a comprehensive strategy, with lifestyle modification as the cornerstone, emphasizing a balanced diet, caloric restriction, and increased physical activity. Pharmacotherapy serves as an adjunct method, with the primary objective of supporting behavioral changes and preventing a decrease in the basal metabolic rate. Despite these options, the long-term success of conservative methods remains limited. Since the late 1990s, bariatric (metabolic) surgery has become the most effective method of treating obesity [3]. Although conservative approaches fail in over 80 % of patients in the long term, metabolic surgery achieves sustained success in more than 80 % of operated individuals [4]. Furthermore, surgical treatment has been confirmed to be more effective than pharmacological therapy alone, even after 5 years of follow-up [5].

Despite significant success in weight reduction and metabolic improvement, obesity patients, including those after bariatric surgery, are still at increased risk of developing additional comorbidities such as type 2 diabetes mellitus, liver steatosis, hypertension, and dyslipidemia. Special attention must be paid to a condition known as sarcopenic obesity, which combines muscle mass loss and strength with increased fat mass, representing a synergistic factor for the progression of metabolic, cardiovascular, and functional disorders [5]. Although there is no universally accepted definition of sarcopenic obesity, sarcopenia itself is clearly defined as the gradual and generalized loss of muscle mass and strength, according to the consensus of the European Working Group on Sarcopenia in Older People (EWGSOP) [6].

The development of sarcopenia is primarily driven by aging, during which a loss of muscle mass of 1 to 2 % per year occurs after the age of 50 [7]. This process is accompanied by a decrease in muscle strength, impaired neuromuscular coordination, and changes in body composition. However, it is not limited to older age groups; alterations in the ratio of muscle to fat mass are also observed in younger obese individuals, significantly contributing to the development of health complications. Numerous studies suggest that obesity and associated insulin resistance can significantly accelerate the onset of sarcopenia, while muscle mass loss itself may reduce energy expenditure and basal metabolism, thus facilitating further weight gain and the progression of obesity [8]. In addition, reduction in muscle mass limits insulin-sensitive tissue, further exacerbating insulin resistance and metabolic dysfunction.

Thus, sarcopenic obesity is associated with an increased risk of developing a variety of serious health conditions, including hypertension [9], increased arterial stiffness [10], dyslipidemia [11], nonalcoholic fatty liver disease (NAFLD) [12], insulin resistance [13], knee osteoarthritis, and osteoporosis with a greater risk of falls and fractures [14]. Furthermore, it is associated with cognitive decline and reduced physical capabilities, severely impacting the quality of life of affected individuals [15].

In recent years, microRNAs (miRNAs) have attracted considerable research interest because of their potential as noninvasive biomarkers. They are stable in body fluids and allow for early detection of cellular changes. In the context of sarcopenia, obesity and their combination, specific miRNA profiles have been identified that correlate with loss of muscle mass, increased fat accumulation, and inflammatory activation [1,6,7].

## Methods

For the purposes of this review article, a literature search was conducted that focused on the relationship between microRNA (miRNA) and sarcopenic obesity. The search was carried out during March and April 2025 in the following databases: PubMed, Scopus, and ScienceDirect (Web of Science). The identified miRNAs were subse-quently examined using specialized biological databases, namely miRBase and miRNet, to gain a deeper under-standing of their biological functions and target gene regulation.

The search strategy involved the use of specific keywords and their combinations: “microRNA”, “miRNA”, “sarcopenic obesity”, “sarcopenia”, and “obesity”.

Priority was given to articles published within the last ten years that directly addressed the relationship between miRNAs and sarcopenic obesity. Only peer-reviewed articles published in English with an available abstract were included in the review.

Based on this search strategy, only five relevant articles were identified that met the criteria mentioned above [16–20].

This low number of publications highlights the fact that the field of miRNA research in the context of sarcopenic obesity remains significantly underexplored, emphasizing the current relevance and research potential of this topic.

### MicroRNA (miRNA)

**Fig. 1:**
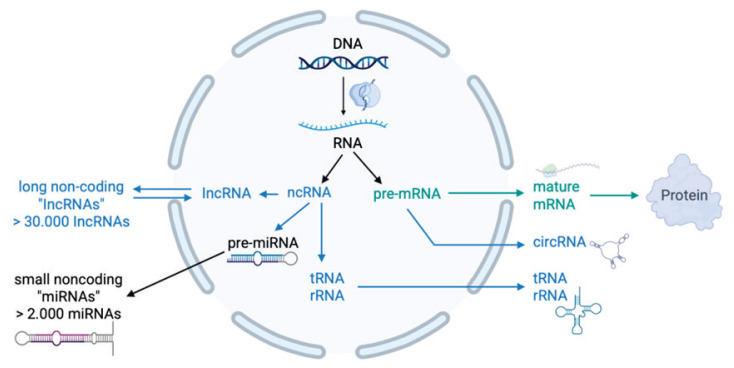
Coding vs. non-coding RNAs in the human genome. circRNA – circular RNA, DNA – deoxyribonucleic acid, lncRNA – long non-coding RNA, miRNA – microRNA, mRNA – messenger RNA, ncRNA – non-coding RNA, pre-miRNA – precursor microRNA, pre-mRNA – precursor messenger RNA, rRNA – ribosomal RNA, RNA – ribonucleic acid, tRNA – transfer RNA.

**Fig. 2:**
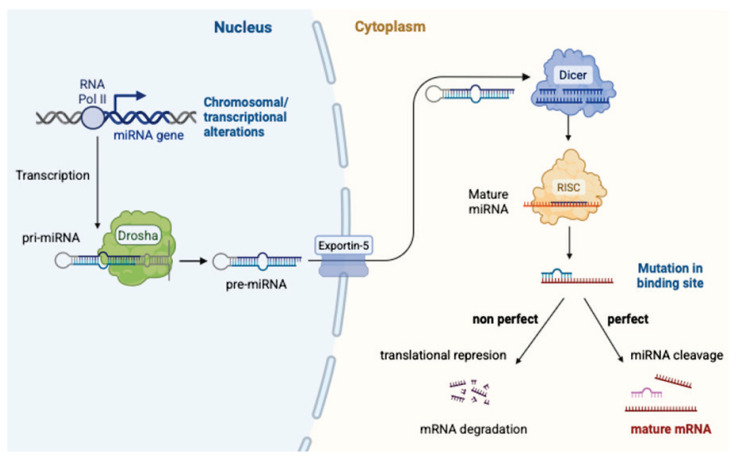
Biogenesis of microRNA. Dicer – RNase III endoribonuclease (processes pre-miRNA into mature miRNA), Drosha – RNase III endonuclease (part of the microprocessor complex that cleaves pri-miRNA into pre-miRNA), Exportin-5 – nuclear export protein (transports pre-miRNA from nucleus to cytoplasm), mRNA – messenger RNA (messenger ribonucleic acid), miRNA – microRNA (micro ribonucleic acid), mature miRNA – mature microRNA, pre-miRNA – precursor microRNA, pri-miRNA – primary microRNA, RISC – RNA-induced silencing complex, RNA – ribonucleic acid, RNA Pol II – RNA polymerase II.

MicroRNAs (miRNAs) are short (18–24 nucleo-tide) noncoding RNA molecules that play a key role in the posttranscriptional regulation of gene expression. They originate from primary transcripts (pri-miRNAs), which are processed by the enzymes Drosha and Dicer and subsequently incorporated into the RNA-induced silencing complex (RISC). Within this complex, miRNAs act on target mRNAs by blocking their translation or inducing their degradation [21–23]. Figure 1 provides an overview of coding and non-coding RNAs within the human genome, highlighting the position of miRNAs among non-coding RNA species. This process is further illustrated in Figure 2, which depicts the key steps of microRNA biogenesis from primary transcripts to their incorporation into the RISC complex.

MiRNAs are involved in a wide range of biological processes, including regulation of cell proliferation, differentiation, apoptosis, glucose and lipid metabolism, and immune responses [24,25]. Their stability in blood, urine, and other body fluids makes them promising biomarkers, and due to their specific regulatory effects, they are also being considered potential therapeutic targets [26,27].

## Analysis and pre-analysis aspects of circulating miRNAs

Reliable analysis of circulating miRNAs requires not only rigorous control of preanalytical factors but also a standardized analytical workflow. The process typically involves total RNA isolation, subsequent reverse transcription of RNA in cDNA, and quantification using real-time PCR (RT-qPCR), or alternatively, next-generation sequencing (NGS) or microarray technologies [47,48]. A critical step is appropriate data normalization, which remains challenging due to the absence of a universally accepted reference marker [44,45].

The preanalytical phase plays a pivotal role in the analysis of circulating miRNA, as does the analysis of laboratory diagnostics. Hemolysis is considered one of the most significant interfering factors in this context, as it results in the massive release of erythrocyte-derived miRNAs, particularly miR-16 and miR-451, which can artificially distort analytical results. To assess the integrity of the sample, the miR-451a/miR-23a ratio has been proposed as a reliable indicator of hemolytic interference [44–46]. Standardization of preanalytical procedures, including the choice of biological material (plasma vs. serum), sampling conditions, time to centrifugation, and sample storage and transport, is essential to ensure both the stability and reproducibility of the results [47,48].

## The significance of individual microRNAs in sarcopenic obesity according to available publications

Dowling *et al*. [16], in their review, identified a total of 24 microRNAs whose expression is altered in both obesity and sarcopenia. Twenty-two of these miRNAs were detected in plasma, one in serum, and one in skeletal muscle. The authors discuss their potential use as biomarkers in the context of sarcopenic obesity. For these 24 miRNAs, they also describe their known biological functions with references to the available literature (Table 1).

The study by Pedraza-Vázquez *et al*. [17] investigated the impact of low-intensity lifelong exercise on changes in miRNA expression during aging and its potential role in the prevention of osteosarcopenic obesity (OSO). In this study, laboratory rats were used to observe the effects of lifelong physical activity on body composition, muscle function, inflammatory status, and expression of specific miRNAs.

Rats were divided into two main groups based on lifestyle – sedentary (SED) and lifelong resistance exercised rats (LRER) – and further subdivided according to age ranges: 8–12 months (young), 12–18 months (middle-aged) and 18–24 months (older). In each age group, miRNA expression was assessed in the gastrocnemius muscle using microarray analysis.

In the 8–12 month group, six common miRNAs were found in both the SED and LRER groups, showing opposite expression trends – up-regulated in SED and down-regulated in LRER.

In the second group (12–18 months), ten common miRNAs were identified. Five of them showed reduced expression in both groups; however, the degree of down-regulation was lower in the exercised group compared to the sedentary rats.

In the 18–24 month group, five miRNAs were identified that were down-regulated in sedentary rats and up-regulated in the exercise group. Detailed information on the identified miRNAs is provided in Table 2.

Chen *et al*. [18], in their review, focused on the main mechanisms by which exercise positively influences body composition, reduces chronic inflammation, improves mitochondrial quality, and regulates hormonal balance. Special attention is paid to the role of myokines and modulation of miRNA expression, which plays a crucial role in the regulation of muscle regeneration, fat metabolism, and inflammatory processes. The article also highlights the need for further research to identify optimal exercise regimens and validate circulating miRNAs as potential biomarkers for sarcopenic obesity.

Regarding miRNAs, the authors point out that the expression of certain miRNAs, such as miR-628-5p, increases with age and its elevated levels affect muscle cell regeneration. Acute resistance exercise in elderly individuals suppresses miR-628-5p expression, thus promoting muscle regeneration. Exercise also stimulates the expression of other miRNAs in adipose tissue, such as miRNA-155-5p, miRNA-329-3p, and miRNA-377-3p, improving lipolysis and improving insulin sensitivity.

Furthermore, the authors note that exercise reduces chronic inflammation by increasing the expression of miRNAs such as miRNA-146d-5p, miRNA-152-3p, miRNA-296-3p, and miRNA-20a-5p, thus decreasing the ratios of pro-inflammatory to anti-inflammatory cytokines.

Rehabilitation exercise in sarcopenic patients increases miRNA-355-5p and miRNA-657 levels, which regulate inflammation and are associated with improved physical function. According to Chen *et al*. [18], miRNAs such as miRNA-23a, miRNA-27a and miRNA-133a, which support muscle regeneration, could play an important role in the diagnosis and monitoring of sarcopenic obesity.

Wilhelmsen *et al*. [19] in their review focused on the crosstalk between adipose tissue and skeletal muscle in the context of aging and obesity, particularly with respect to the development of sarcopenia and sarcopenic obesity.

They demonstrated how adipose tissue, now recognized as an endocrine organ, influences muscle mass and function through the secretion of various factors, including cytokines (e.g., resistin, adiponectin, leptin, lipocalin-2, myostatin), myokines, adipokines, long noncoding RNAs (lncRNAs), and miRNAs.

MiRNAs are key regulators of gene expression and play a crucial role in maintaining muscle mass, muscle cell differentiation, and adipose tissue metabolism, especially in the context of aging and obesity.

miRNA-130b, produced by adipocytes during adipogenesis and elevated in obesity, as well as miRNA-31, miRNA-223, and miRNA-33a, are consi-dered potential mediators of the adverse effects of obesity on muscle mass regulation.

MiRNA-133a and miRNA-133b, which typically promote myoblast proliferation, along with miRNA-1 and miRNA-206, which support myogenesis, are influenced by obesity and aging. Therefore, targeted manipulation of specific miRNAs could represent a novel therapeutic strategy to combat muscle mass loss and to treat sarcopenic obesity.

Papadopoulos *et al*. [20] investigated the key role of miRNA-155 in the pathogenesis of type 2 diabetes mellitus (DM2), which is closely linked to sarcopenia and obesity. MiRNA-155 is a small noncoding RNA molecule that regulates the expression of more than 241 genes and significantly affects insulin signaling, inflammatory pathways, oxidative stress, and the regulation of the angiotensin II type 1 receptor (AT1R) within the renin-angiotensin-aldosterone system (RAAS). The authors report that miRNA-155 levels are significantly decreased in DM2, obesity, and sarcopenia, leading to worsening insulin resistance and disease progression.

A decrease in miRNA-155 results in the deregulation of its key target genes, which contributes to impaired glucose homeostasis, increased inflammation, oxidative stress, and pancreatic β-cell loss.

Specifically, reduction in miRNA-155 allows excessive activation of RAAS through angiotensin II through the AT1R receptor, exacerbating adverse effects on the vasculature, kidneys, and metabolism.

The article emphasizes that future therapies could focus on the targeted administration of synthetic miRNA-155 analogs to improve insulin sensitivity, protect β-cells, and attenuate the adverse effects of RAAS activation in DM2.

## Genetics and molecular mechanisms regulating miRNA in sarcopenic obesity

Sarcopenic obesity represents the result of a complex interaction between genetic factors and environmental influences such as nutrition, physical activity, and aging. A significant component of this interaction involves single nucleotide polymorphisms (SNPs), which may influence the expression of microRNAs (miRNAs) and therefore modulate their regulatory effects on gene expression [22,23,34,35].

One of the most extensively studied genetic polymorphisms in the context of muscle function is the R577X variant of the ACTN3 gene, which has been associated with reduced muscle strength. This genetic variant may also affect the expression of miRNA-1 and miRNA-133, two miRNAs that play essential roles in myogenesis, the process of muscle fiber formation and regeneration [34–36,42].

The results of genome-wide association studies (GWAS) have identified several additional genetic variants associated with obesity risk and metabolic disorders, particularly within the FTO, MC4R, NUDT3 and GPD1L genes. These genes are involved in adipogenesis and the regulation of energy metabolism, their activity being partially regulated by specific miRNAs [24,37].

Special attention should be paid to miRNAs directly involved in muscle metabolism, particularly miRNA-486, miRNA-1, miRNA-133a and miRNA-206. The expression of these miRNAs varies depending on the individual’s genetic background and level of physical activity, reflecting their sensitivity to exercise-induced stress and adaptive responses [8,40–42].

In experimental studies, for example, Alexander *et al*. [40] described the effect of miRNA-486 on the DOCK3/PTEN/AKT signaling pathway, which is critical for muscle cell growth and survival [40]. Likewise, Li *et al*. [38] demonstrated that circulating miRNAs exhibit significant changes in expression in response to physical activity, underscoring their potential as biomarkers of muscular load and adaptation [38,40].

## Mutual regulation of microRNAs and myokines in the pathogenesis of sarcopenic obesity

In recent years, the interaction between microRNAs (miRNAs) and muscle-derived cytokines, known as myokines, has been recognized as a key factor in the pathogenesis of sarcopenic obesity. Myokines are bioactive proteins secreted by skeletal muscle tissue in response to contraction or metabolic stress. Among the most studied are irisin, myonectin, interleukin-6 (IL-6), myostatin, and insulin-like growth factor 1 (IGF-1). In individuals with sarcopenia, there is a reduction in proteoanabolic myokine production (eg IGF-1, IL-15) and a simultaneous increase in catabolic factors such as myostatin, which inhibits muscle growth and promotes lipogenesis [21–25]. These alterations significantly influence the expression of muscle-specific miRNAs (eg miRNA-1, miRNA-133, miRNA-206), which regulate the proliferation, differentiation, and regeneration of muscle cells [26–28,42].

In contrast, certain miRNAs influence the production and signaling of myokines. For example, miRNA-486 enhances IGF-1R expression and activates the PI3K/AKT signaling pathway; miRNA-206 suppresses myostatin expression; and miRNA-21 and miRNA-155 promote inflammatory responses that attenuate the anabolic effects of myokines such as IL-15 [26–30].

Dysregulation of this miRNA-myokine network leads to an imbalance between anabolic and catabolic signals, resulting in progressive muscle loss accompanied by fat accumulation, hallmarks of sarcopenic obesity.

Integrating knowledge about miRNAs and myokines provides a novel approach to understanding interorgan signaling between skeletal muscle and adipose tissue. It also opens new avenues for multimodal diagnostics and targeted therapeutic strategies in the management of sarcopenic obesity.

## Conclusions

MicroRNAs (miRNAs) and myokines represent two key classes of molecules involved in the regulation of muscle metabolism, immune function, and body composition. Their mutual interaction forms a complex regulatory network that significantly contributes to the development and progression of sarcopenic obesity. Dysregulation of these systems leads to chronic inflammation, impaired muscle regeneration, and fat accumulation within muscle tissue [21–28].

Research on miRNAs in the context of sarcopenic obesity is still in its early stages, as reflected by the very limited number of available studies. However, existing data already suggest that miRNAs hold considerable potential not only as noninvasive biomarkers that enable early diagnosis but also as therapeutic targets capable of influencing disease progression. Due to their stability in body fluids, tissue-specific expression, and the ability to reflect metabolic status in muscle and adipose tissue, miRNAs could be used to detect at-risk individuals with sarcopenic obesity and to monitor the effectiveness of therapeutic interventions [16–20].

In diagnostics, the analysis of circulating miRNAs could facilitate the early detection of muscle atrophy and metabolic disturbances, prediction of the progression of sarcopenic obesity, and monitoring of treatment response [16–20].

In terms of therapy, modulation of specific miRNA expression (e.g., *via* miRNA mimics or inhibitors), targeted interference with pathogenic signaling pathways associated with muscle wasting, insulin resistance, and inflammation, as well as personalized treatment approaches based on individual miRNA profiles, represent promising strategies [19,20].

Therefore, future research should focus on the detection of miRNAs in patients with confirmed sarcopenic obesity, followed by the identification of candidate miRNAs for the diagnosis and therapy of this condition. In this context, Table 3 was developed, summarizing the most relevant miRNAs that, according to the available literature, have the greatest potential for clinical application in the diagnosis, monitoring and treatment of sarcopenic obesity. However, it is important not to overlook the need to conduct clinical validations of miRNAs as biomarkers, as well as the development of therapeutic strategies based on modulation of key miRNA expression, ideally in combination with pharmacotherapy and physical activity [18].

The targeted integration of miRNAs into clinical practice could fundamentally change the approach to the prevention, diagnosis, and treatment of sarcopenic obesity and represents one of the most promising avenues of personalized medicine in this field [16–20].

## Figures and Tables

**Table 1:** miRNA associated with sarcopenic obesity according to Dowling *et al*. [16].

N.	miRNA	Expression	Function	Analyzed material
1	miRNA-106a-5p	↑	Inhibits myogenesis	Plasma
2	miRNA-18b-5p	↑	Targets IGF-1, suppresses activation of p-AKT, p-MEK, and p-ERK1/2, involved in inflammatory pathways	Plasma
3	miRNA-193b-5p	↑	Regulates the cell cycle and proliferation, negatively correlates with BMI and blood glucose levels	Plasma
4	miRNA-197-3p	↑	Inhibits GIP and GLP-1 production, influences glucose metabolism; elevated after intensive resistance training in young adults	Plasma
5	miRNA-199a-5p	↓	Targets SIRT1 and GLUT4, regulates glucose uptake, and promotes apoptosis and ROS formation	Plasma
6	miRNA-483-3p	↑	Targets IGF-1, inhibits myoblast proliferation, promotes apoptosis in hyperglycemic cardiomyocytes	Plasma
7	miRNA-499	↓	Associated with the slow-twitch muscle fiber phenotype in humans, a regulator of mitochondrial function in myocytes; expression is increased in diabetic and obese mice	Plasma
8	miRNA-550a-3p	↑	Limited studies regarding muscle/obesity; associated with bone structure parameters	Plasma
9	miRNA-576-5p	↑	Function in the context of muscle/obesity remains unclear	Plasma
10	miRNA-589-5p	↑	Decreased after TGF-β stimulation; function in muscle/obesity remains unclear	Plasma
11	miRNA-92a-3p	↑	Systematically reduced after bariatric surgery; influences brown fat differentiation and mitochondrial function	Plasma
12	miRNA-1224-5p	↑	Targets AMPKα1; contributes to lipid accumulation in the liver	Plasma
13	miRNA-1246	↑	Elevated in diabetic nephropathy; positively correlates with BMI	Plasma
14	miRNA-145-5p	↑	Elevated with high-fat diet consumption; limited studies in obesity/sarcopenia	Plasma
15	miRNA-196a-5p	↑	High expression in myoblasts; suppresses mitochondrial biogenesis	Plasma
16	miRNA-296a-3p	↑	Decreased expression of miRNA-296-3p promotes cell proliferation	Plasma
17	miRNA-29b-2-5p	↑	Targets STAT3 in fibroblast cell lines; limited studies in the context of muscle/obesity	Plasma
18	miRNA-301b-3p	↓	Regulates myogenic differentiation *via* Rb1cc1	Plasma
19	miRNA-378c	↑	Limited studies; function in the context of muscle/obesity remains unclear	Plasma
20	miRNA-4732-5p	↑	Limited studies; function in the context of muscle/obesity remains unclear	Plasma
21	miRNA-487a-3p	↓	Limited studies; function in the context of muscle/obesity remains unclear	Plasma
22	miRNA-766-3p	↑	Increased in older adults, influenced by training	Plasma
23	miRNA-23a-3p	↑	Elevated after resistance or endurance exercise; protects muscle from atrophy	Serum
24	miRNA-424-5p	↑	Targets IGF-1 in mice and human myocytes, influences protein synthesis and insulin signaling	Skeletal muscle

**Table 2:** Overview of miRNA by laboratory rat grouping and expression patterns. Pedraza-Vázquez *et al*. [17].

Group (Age)	Number of Shared miRNA	Regulation Pattern	List of miRNA
8–12 months	6	Upregulated in sedentary, downregulated in exercised	rno-miRNA-134-5p, rno-miRNA-678, rno-miRNA-23a-5p, rno-miRNA-125a-5p, rno-miRNA-6332, rno-miRNA-375
12–18 months	5	Upregulated in sedentary, downregulated in exercised	rno-miRNA-30e-5p, rno-miRNA-1839-5p, rno-miRNA-194-5p, rno-miRNA-10b-5p, rno-miRNA-497-5p
12–18 months	5	Downregulated in both groups	rno-miRNA-494-3p, rno-miRNA-127-3p, rno-miRNA-672-5p, rno-miRNA-32-3p, rno-miRNA-122-5p
18–24 months	5	Upregulated in exercised, downregulated in sedentary	rno-miRNA-152-3p, rno-miRNA-146a-5p, rno-miRNA-1839-5p, rno-miRNA-296-3p, rno-miRNA-20a-5p

**Table 3:** Overview of candidate miRNAs with potential use in the diagnosis, monitoring, and therapy of sarcopenic obesity according to available publications.

miRNA	Regulation pattern	Mechanism/Signaling pathways	Potential use	Reference
miR-155	Decreased expression in SO, obesity and T2DM; increased with exercise	RAAS (AT1R), insulin signaling, inflammation	Diagnosis, monitoring, therapeutic target	Papadopoulos *et al*. [20]; Chen *et al*. [18]
miR-486-5p	Decreased expression in SO, influenced by age and genetics	Targets IGF-1R, DOCK3/PTEN/AKT signaling pathway	Diagnosis, therapeutic target	Alexander *et al*. [40]; Wilhelmsen *et al*. [19]
miR-1, miR-133a, miR-206	Dysregulation associated with age, obesity and training	Myogenesis, proliferation, muscle regeneration	Diagnosis, monitoring of therapeutic response	Wilhelmsen *et al*. [19]; McCarthy [27]; Chen *et al*. [18]; Koutsoulidou *et al*. [43]
miR-23a	Increased expression with exercise, protects against atrophy	IGF-1/AKT, muscle regeneration	Monitoring regeneration and effect of exercise	Chen *et al*. [18]; Dowling *et al*. [16]
miR-145-5p	Increased expression after fat intake	Regulation of lipid metabolism	Diagnostic marker of metabolic stress	Dowling *et al*. [16]
miR-1246	Increased expression in obesity, positive correlation with BMI	Lipid metabolism, inflammatory pathways	Diagnostic marker	Dowling *et al*. [16]
miR-378	Increased expression in obesity	Mitochondria, energy homeostasis	Therapeutic target (anti-obesity effect)	Carrer *et al*. [31]; Dowling *et al*. [16]
miR-33a	Dysregulation in obesity	Cholesterol and lipid metabolism	Risk stratification of SO phenotype	Wilhelmsen *et al*. [19]
miR-223	Increased expression in obesity	Regulation of inflammation	Marker of inflammatory response and SO	Wilhelmsen *et al*. [19]
miR-628-5p	Increased expression with age, decreased with exercise	Muscle cell regeneration	Marker of aging and rehabilitation response	Chen *et al*. [18]
miR-146d-5p, miR-152-3p, miR-296-3p, miR-20a-5p	Increased expression with exercise	Anti-inflammatory signaling pathways	Monitoring the anti-inflammatory effect of exercise	Chen *et al*. [18]
miR-657, miR-355-5p	Increased expression after rehabilitation	Regulation of inflammation, functional improvement	Monitoring rehabilitation response	Chen *et al*. [18]

## References

[1] Walpole SC, Prieto-Merino D, Edwards P, Cleland J, Stevens G, Roberts I (2012). The weight of nations: an estimation of adult human biomass. BMC Public Health.

[2] Matoulek M, Svačina Š, Lajka J (2010). Výskyt obezity a jejích komplikací v České republice. Vnitr Lek.

[3] Buchwald H, Avidor Y, Braunwald E, Jensen MD, Pories W, Fahrbach K, Schoelles K (2004). Bariatric surgery: a systematic review and meta-analysis. JAMA.

[4] Fried M, Yumuk V, Oppert JM, Scopinaro N, Torres A, Weiner R, Yashkov Y (2014). Interdisciplinary European guidelines on metabolic and bariatric surgery. Obes Surg.

[5] Schauer PR, Bhatt DL, Kirwan JP, Wolski K, Aminian A, Brethauer SA, Navaneethan SD (2017). Bariatric Surgery versus Intensive Medical Therapy for Diabetes - 5-Year Outcomes. N Engl J Med.

[6] Cruz-Jentoft AJ, Bahat G, Bauer J, Boirie Y, Bruyère O, Cederholm T, Cooper C (2019). Sarcopenia: Revised European consensus on definition and diagnosis. Age Ageing.

[7] Choi KM (2013). Sarcopenia and Sarcopenic Obesity. Endocrinol Metab.

[8] Kohara K (2014). Sarcopenic obesity in aging population: current status and future directions for research. Endocrine.

[9] Park SH, Park JH, Song PS, Kim DK, Kim KH, Seol SH, Kim HK (2013). Sarcopenic obesity as an independent risk factor of hypertension. J Am Soc Hypertens.

[10] Kohara K, Ochi M, Tabara Y, Nagai T, Igase M, Miki T (2012). Arterial stiffness in sarcopenic visceral obesity in the elderly: J-SHIPP study. Int J Cardiol.

[11] Baek SJ, Nam GE, Han KD, Choi SW, Jung SW, Bok AR, Kim YH (2014). Sarcopenia and sarcopenic obesity and their association with dyslipidemia in Korean elderly men: the 2008–2010 Korea National Health and Nutrition Examination Survey. J Endocrinol Invest.

[12] Hong HC, Hwang SY, Choi HY, Yoo HJ, Seo JA, Kim SG, Kim NH (2014). Relationship between sarcopenia and nonalcoholic fatty liver disease: The Korean Sarcopenic Obesity Study. Hepatology.

[13] Kim TN, Park MS, Lim KI, Choi HY, Yang SJ, Yoo HJ, Kang HJ (2013). Relationships between sarcopenic obesity and insulin resistance, inflammation, and vitamin D status: the Korean Sarcopenic Obesity Study. Clin Endocrinol (Oxf).

[14] Waters DL, Hale L, Grant AM, Herbison P, Goulding A (2010). Osteoporosis and gait and balance disturbances in older sarcopenic obese New Zealanders. Osteoporos Int.

[15] Baumgartner RN, Wayne SJ, Waters DL, Janssen I, Gallagher D, Morley JE (2004). Sarcopenic Obesity Predicts Instrumental Activities of Daily Living Disability in the Elderly. Obes Res.

[16] Dowling L, Duseja A, Vilaca T, Walsh JS, Goljanek-Whysall K (2022). MicroRNAs in obesity, sarcopenia, and commonalities for sarcopenic obesity: a systematic review. J Cachexia Sarcopenia Muscle.

[17] Pedraza-Vázquez G, Mena-Montes B, Hernández-Álvarez D, Gómez-Verjan JC, Toledo-Pérez R, López-Teros MT, Königsberg M (2023). A low-intensity lifelong exercise routine changes miRNA expression in aging and prevents osteosarcopenic obesity by modulating inflammation. Arch Gerontol Geriatr.

[18] Chen J, Jia S, Guo C, Fan Z, Yan W, Dong K (2024). Research Progress on the Effect and Mechanism of Exercise Intervention on Sarcopenia Obesity. Clin Interv Aging.

[19] Wilhelmsen A, Tsintzas K, Jones SW (2021). Recent advances and future avenues in understanding the role of adipose tissue cross talk in mediating skeletal muscle mass and function with ageing. GeroScience.

[20] Papadopoulos KI, Papadopoulou A, Aw TC (2023). MicroRNA-155 mediates endogenous angiotensin II type 1 receptor regulation: implications for innovative type 2 diabetes mellitus management. World J Diabetes.

[21] Grieb A, Schmitt A, Fragasso A, Widmann M, Maturana FM, Burgstahler C, Erz G (2023). Skeletal Muscle MicroRNA Patterns in Response to Exercise. Biomolecules.

[22] Mazurkiewicz Ł, Czernikiewicz K, Grygiel-Górniak B (2024). Immunogenetic Aspects of Sarcopenic Obesity. Genes.

[23] Khanal P, Williams AG, He L, Stebbings GK, Onambele-Pearson GL, Thomis M, Degens H, Morse CI (2021). Sarcopenia, Obesity, and Sarcopenic Obesity: Relationship with Skeletal Muscle Phenotypes and SNPs. J Clin Med.

[24] Jin H, Yoo HJ, Kim YA, Lee JH, Lee Y, Kwon S-H, Seo YJ (2022). Unveiling genetic variants for age-related sarcopenia: GWAS in Korean cohorts. Sci Rep.

[25] Teodori L, Costa A, Campanella L, Albertini MC (2019). Immuno-Related MicroRNAs and Muscle Atrophy. Front Physiol.

[26] van Rooij E, Sutherland LB, Qi X, Richardson JA, Hill J, Olson EN (2007). Control of stress-dependent cardiac growth and gene expression by a microRNA. Science.

[27] McCarthy JJ (2008). MicroRNA-206: the skeletal muscle-specific myomiR. Biochim Biophys Acta.

[28] Chen J-F, Mandel EM, Thomson JM, Wu Q, Callis TE, Hammond SM, Conlon FL, Wang D-Y (2006). The role of microRNA-1 and microRNA-133 in skeletal muscle proliferation and differentiation. Nat Genet.

[29] Nielsen S, Scheele C, Yfanti C, Åkerström T, Nielsen AR, Pedersen BK, Laye MJ (2010). Muscle specific microRNAs are regulated by endurance exercise in human skeletal muscle. J Physiol.

[30] Caria ACI, Nonaka CKV, Pereira CS, Soares MBP, Macambira SG, de Freitas Souza BS (2018). Exercise Training-Induced Changes in MicroRNAs: Beneficial Regulatory Effects in Hypertension, Type 2 Diabetes, and Obesity. Int J Mol Sci.

[31] Carrer M, Liu N, Grueter CE, Williams AH, Frisard MI, Hulver MW, Bassel-Duby R, Olson EN (2012). Control of mitochondrial metabolism and systemic energy homeostasis by microRNAs 378 and 378*. Proc Natl Acad Sci U S A.

[32] Pan D, Mao C, Quattrochi B, Friedline RH, Zhu LJ, Jung DY, Kim JK, Lewis B, Wang YX (2014). MicroRNA-378 controls classical brown fat expansion to counteract obesity. Nat Commun.

[33] Najafi-Shoushtari SH, Kristo F, Li Y, Shioda T, Cohen DE, Gerszten RE, Näär AM (2010). MicroRNA-33 and the SREBP host genes cooperate to control cholesterol homeostasis. Science.

[34] Pickering C, Kiely J (2017). ACTN3: More than just a gene for speed. Front Physiol.

[35] Cho J, Lee I, Kang H (2017). ACTN3 Gene and Susceptibility to Sarcopenia and Osteoporotic Status in Older Korean Adults. BioMed Research International.

[36] Del Coso J, Hiam D, Houweling P, Pérez LM, Eynon N, Lucía A (2019). More than a ‘speed gene’: ACTN3 R577X genotype, trainability, muscle damage, and the risk for injuries. Eur J Appl Physiol.

[37] Jin H, Yoo HJ, Kim YA, Lee JH, Lee Y, Kwon SH, Seo YJ (2022). Unveiling genetic variants for age-related sarcopenia by conducting a genome-wide association study on Korean cohorts. Sci Rep.

[38] Li Y, Yao M, Zhou Q, Cheng Y, Che L, Xu J, Xiao J, Shen Z, Bei Y (2018). Dynamic regulation of circulating microRNAs during acute exercise and long-term exercise training in basketball athletes. Front Physiol.

[39] Pedersen BK (2009). Muscle as an endocrine organ: IL-6 and other myokines. J Appl Physiol (1985).

[40] Alexander MS, Casar JC, Motohashi N, Vieira NM, Eisenberg I, Marshall JL, Gasperini MJ (2014). MicroRNA-486-dependent modulation of DOCK3/PTEN/AKT signaling pathways improves muscular dystrophy-associated symptoms. J Clin Invest.

[41] Elia L, Quintavalle M, Zhang J, Contu R, Cossu L, Latronico MVG, Peterson KL (2009). The knockout of miR-143 and -145 alters smooth muscle cell maintenance and vascular homeostasis in mice: correlates with human disease. Cell Death Differ.

[42] Kim HK, Lee YS, Sivaprasad U, Malhotra A, Dutta A (2006). Muscle-specific microRNA miR-206 promotes muscle differentiation. J Cell Biol.

[43] Koutsoulidou A, Mastroyiannopoulos NP, Furling D, Uney JB, Phylactou LA (2011). Expression of miR-1, miR-133a, miR-133b and miR-206 increases during development of human skeletal muscle. BMC Dev Biol.

[44] Kirschner MB, Kao SC, Edelman JJ, Armstrong NJ, Vallely MP, van Zandwijk N, Reid G (2011). Haemolysis during sample preparation alters microRNA content of plasma. PLoS One.

[45] Kirschner MB, Edelman JJ, Kao SC, Vallely MP, van Zandwijk N, Reid G (2013). The Impact of Hemolysis on Cell-Free microRNA Biomarkers. Front Genet.

[46] Shah JS, Soon PS, Marsh DJ (2016). Comparison of Methodologies to Detect Low Levels of Hemolysis in Serum for Accurate Assessment of Serum microRNAs. PLoS One.

[47] Becker N, Lockwood CM (2013). Pre-analytical variables in miRNA analysis. Clin Biochem.

[48] Zendjabil M (2024). Preanalytical, analytical and postanalytical considerations in circulating microRNAs measurement. Biochem Med (Zagreb).

